# NMR Study on Small Proteins from *Helicobacter pylori* for Antibiotic Target Discovery: A Review

**DOI:** 10.3390/molecules181113410

**Published:** 2013-10-30

**Authors:** Su-Jin Kang, Do-Hee Kim, Bong-Jin Lee

**Affiliations:** Research Institute of Pharmaceutical Sciences, College of Pharmacy, Seoul National University, Seoul 151-742, Korea

**Keywords:** *Helicobacter pylori*, antimicrobial peptides (AMPs), antibiotics, nuclear magnetic resonance (NMR), structure-activity relationship (SAR)

## Abstract

Due to the widespread and increasing appearance of antibiotic resistance, a new strategy is needed for developing novel antibiotics. Especially, there are no specific antibiotics for *Helicobacter pylori* (*H. pylori*). *H. pylori* are bacteria that live in the stomach and are related to many serious gastric problems such as peptic ulcers, chronic gastritis, mucosa-associated lymphoid tissue lymphoma, and gastric cancer. Because of its importance as a human pathogen, it’s worth studying the structure and function of the proteins from *H. pylori*. After the sequencing of the *H. pylori* strain 26695 in 1997, more than 1,600 genes were identified from *H. pylori*. Until now, the structures of 334 proteins from *H. pylori* have been determined. Among them, 309 structures were determined by X-ray crystallography and 25 structures by Nuclear Magnetic Resonance (NMR), respectively. Overall, the structures of large proteins were determined by X-ray crystallography and those of small proteins by NMR. In our lab, we have studied the structural and functional characteristics of small proteins from *H. pylori*. In this review, 25 NMR structures of *H. pylori* proteins will be introduced and their structure-function relationships will be discussed.

## 1. Development of Antibiotics

Antibiotics are agents that suppress bacterial growth or kill bacteria. The term “antibiotic” was introduced in 1942 by Waksman and his collaborators. At first, antibiotics included only microbially-originated chemical substances, which inhibit the growth or the metabolic activities of bacteria and other micro-organisms. Recently, the concept of antibiotics has been enlarged to include anti-fungals and other compounds [1].

Antibiotics can be classified according to how they affect the cellular component or system, and to how they work, namely by killing the cell (bactericidal drugs) or stopping cell-growth (bacteriostatic drugs). Even though bacterial and human cells share a lot of the same characteristics, there are differences that make antibiotics selectively toxic and they can deliver damage to the pathogen without causing harm to host cells, namely human cells. DNA, RNA, cell walls or the protein synthesis mechanisms of the bacteria are mostly used as antibiotics targets [2,3].

Methods for antibiotic discovery have been developed over the years. In the initial period, a synthetic antibacterial drug like prontosil, for which the active ingredient is a sulfonamide, was used. This drug is classified as a sulfa drug. Up to the 1980s, beginning with the discovery of penicillin by Fleming, antibiotics had been discovered by screening natural products (NP) such as the whole cell fermentation broths of cultured organisms. With this method, most of the major antibiotics used until now such as the aminoglycosides, cephalosporins, chloramphenicol, tetracyclines, macrolides and glycopeptides have been discovered. However, with the NP screening method it has become increasingly difficult to find novel ‘magic bullets’. In mid-1980s, chemical modification of a useful but old antibacterial agent introduced the fluoroquinolone era of antibiotics. Since then, various antibiotic analogs have appeared [4]. From the 1990s, in an effort to identify new classes of antibiotics, bacterial proteins have been used as the core of novel antibiotic targets, and high-throughput (HT) screening based on the information provided by bacterial genomics was developed. HT screening combined with chemical libraries has found a lot of inhibitors and lead compounds potentially useful as new antibiotics [5]. Bacterial proteins identified as potential targets have been studied by X-ray crystallography and NMR to elucidate the active site for *de novo* drug design. Recently, structure-based drug discovery (SBDD) has become most efficient tool to identify novel antibiotics [6].

Bacteria have evolved to adapt to the environment. Through this phenomenon, bacteria have developed resistance to antibiotics. Consequently, “super bugs” have been emerged and can cause epidemics. Recently, the appearance of super bugs from *Vibrio cholerea* [7] and community-acquired methicillin-resistance *Staphylococcus aureus* (CM-MRSA) has been one of the major problems to overcome in pharmaceutics [8,9].

## 2. *Helicobacter pylori* as Antibiotics Target Discovery

*Helicobacter pylori* (*H. pylori*) are Gram-negative and spiral shaped bacteria that live in the stomach and duodenum (the section of intestine just below stomach) [10,11,12]. *H. pylori* was first found in humans in 1906 [13] and successfully cultured in 1983 from gastric biopsies by Marshall and Warren [14], who jointly won the 2005 Nobel prize in Physiology or Medicine for their work on *H. pylori*. According to the estimates of the WHO published in 1994, *H. pylori* infects almost half of the World’s population and are a major cause of gastric inflammation and peptic ulcer diseases [15,16]. The presence of the bacterium in the gastric mucosa is associated with chronic active gastritis and is implicated in more severe gastric diseases, including chronic atrophic gastritis (a precursor of gastric carcinomas), peptic ulceration, and mucosa-associated lymphoid tissue lymphomas [17,18,19]. In addition, the risk developing into gastric cancer is related to the change in the stomach from a *H. pylori* infection [20]. *H. pylori* colonize the human gastric mucosa and persist in the stomach of patients with or without the clinical symptoms over the entire life time of the patients. *H. pylori* are highly adapted to survive in the extreme acidic environment in the human stomach and can penetrate the mucous layer [16]. Systemic understanding of the mechanism underlying inflammation is important to find new antibiotics specific for *H. pylori* [21].

Over the past few decades, medical therapy for *H. pylori* infection has relied on a proton pump inhibitor (PPI) mixed with two antibiotics for two weeks [22,23,24]. Current treatments include omeprazole as a PPI and amoxicillin, tetracycline or metronidazole as antibiotics [25]. Unfortunately, according to recent reports, the efficacy of the treatment has dropped below 80%, mainly due to antibiotic resistance, lack of patient compliance, side-effects of the antibiotics and high cost of treatment [24]. Thus, much effort has been focused on discovering alternative treatment or prevention strategies such as vaccines or immunotherapy [26].

**Figure 1:**
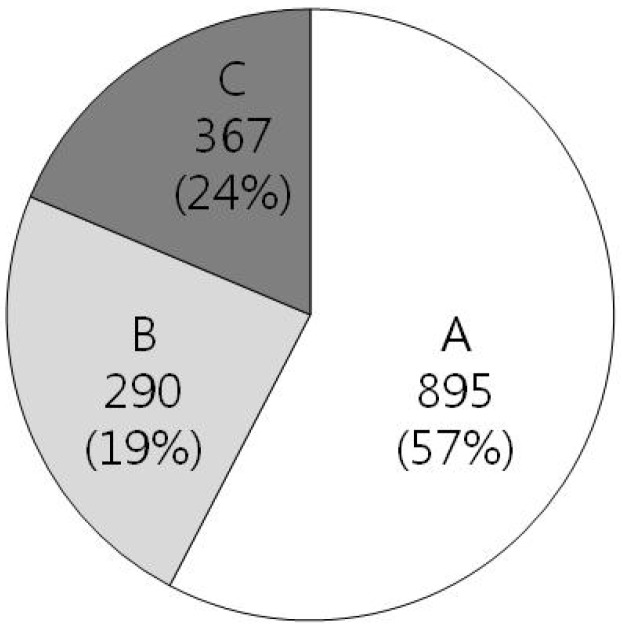
Genomic composition of *H. pylori* strain 29965 (total 1,552 ORFs). A includes genes which have orthologs of known function, B include genes which have orthologs of unknown function, and C includes *H. pylori* specific genes.

Recently, after a database of *H. pylori* genes essential for cell viability was established, useful information to find excellent targets for new antibiotic drugs has been revealed. The genome of *H. pylori* has been fully sequenced for two prototype strains: strain 29965 in 1997 [10] and strain J99 in 1999 [27]. Especially, in the chromosome of strain 26695, 1,552 open reading frames (ORFs) were identified as shown in Figure 1. Among them, only 57% of the ORFs have homologs with genes of known function in other organisms, and approximately 43% of the ORFs still remain as unknown or *H. pylori*-specific genes. Until now, more than 1,600 genes have been identified from *H. pylori*, representatively as VacA [28] and CagA [29].

In order to understand how *H. pylori* work as a pathogen, the structural study of proteins is very important because protein function is highly related to the 3-dimensional (3D) structures of proteins [30]. In the Protein Data Bank (PDB), the structures of 334 proteins from *H. pylori* have been reported as of 6 February 2013. Among them, 309 structures have been determined by X-ray crystallography and 25 structures by Nuclear Magnetic Resonance (NMR), respectively. Usually, the structures of large proteins are determined by X-ray crystallography and those of small proteins by NMR spectroscopy.

Because of its importance as a human pathogen, understanding the mechanism of *H. pylori* inflammation is the most important part in order to discover effective and specific antibiotics for *H. pylori*. In this regard, the structural and functional study of *H. pylori* proteins should be the most important point for developing antibiotics and can provide clues to help cope with new antibiotic-resistant bacteria. Because structural studies on *H. pylori* proteins are very important for structure based drug discovery, 25 NMR structures of *H. pylori* will be introduced and discussed in this review.

## 3. Structures of 25 NMR Proteins from *H. pylori*

**Figure 2:**
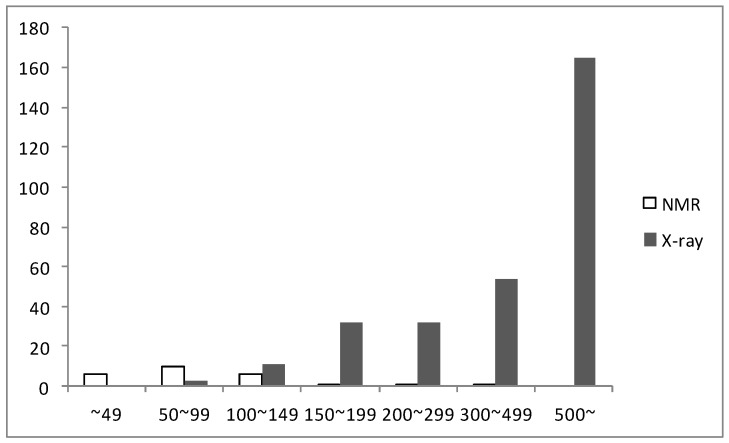
The number of structures determined by X-ray and NMR according to the size.

Until now, more than 1,600 genes have been found from *H. pylori*. After determining the first *H. pylori* protein structure by X-ray crystallography in 2001 (PDB ID: 1G6O) [31], the structures of 334 proteins from *H. pylori* have been determined using either X-ray crystallography or NMR and deposited in the PDB as 6 February 2013. Of these structures, 309 structures were determined by X-ray crystallography and only 25 structures (7.5%) by NMR. X-ray crystallography was typically used for proteins with a large molecular weight and NMR was used for ones with a small molecular weight, as shown in Figure 2. In the case of X-ray crystallography, more than half of the determined structures had over 500 amino acid residues, that is, these proteins have large molecular weights. While NMR could not yet determine the structure of any *H. pylori* protein with over 500 amino acid residues, however, NMR was much more useful for the structure determination of small proteins from *H. pylori*. In our laboratory, we have studied the structural and functional characteristics of small proteins from *H. pylori* using NMR.

**Figure 3:**
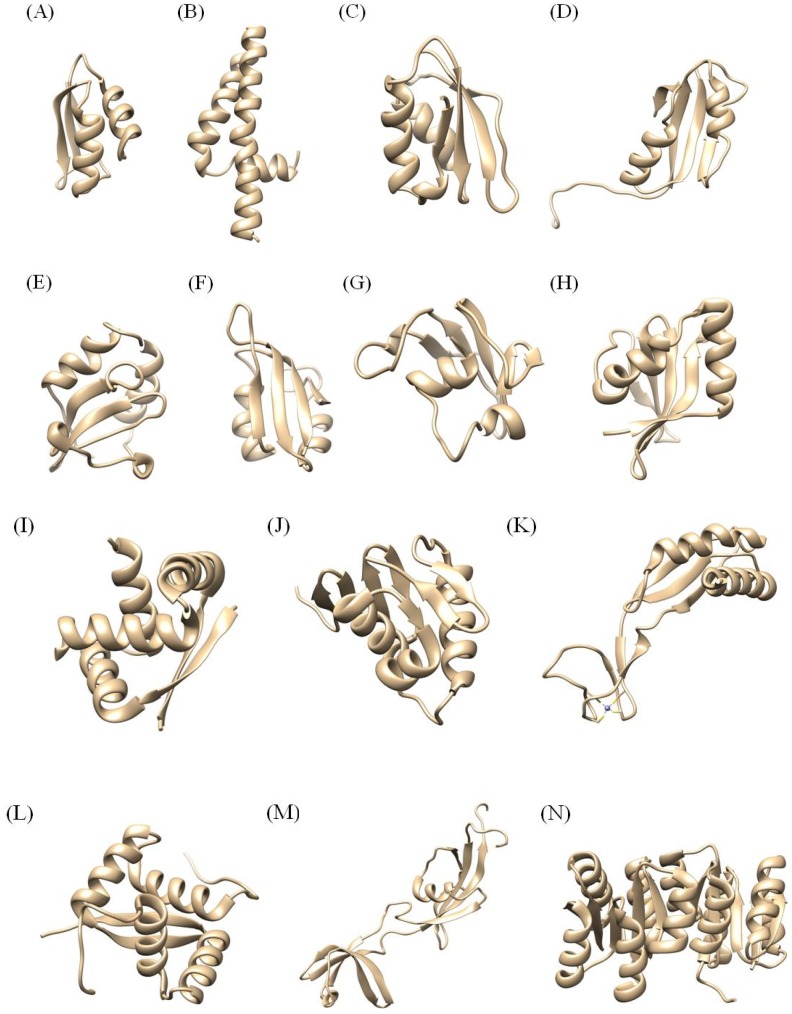
Structures of *H. pylori* proteins determined by NMR. (**A**) 1YG0 (HP1073), (**B**) 1ZHC (HP1242), (**C**) 2LXR (HP1264), (**D**) 2H9Z (HP0495), (**E**) 1Z8M (HP0894), (**F**) 2KI2 (HP0827), (**G**) 2K6P (HP1423), (**H**) 2QTR (HP0892), (**I**) 1X93 (HP0222), (**J**) 2K4J, (**K**) 2KDK, (**L**) 2K1O (HP0564), (**M**) 2KR7 (HP1123), (**N**) 2HQO (HP1043).

Figure 3 shows several structures of *H. pylori* proteins which have been determined until now. Here, 23 NMR structures from *H. pylori* among the 25 in Table 1 will be discussed because 2JOQ and 2K0Z don’t have any structural information and published reference papers.

**Table 1:** Twenty NMR structures of *H. pylori* proteins. All data were collected from the PDB database on 6 February 2013.

	PDB ID	Macromolecule Name	No. of AA	Function	Year
1	1P5K	-	19	ribosome	2003
2	1P5L	-	19	ribosome	2003
3	1P0G	-	19	ribosome	2003
4	1P0J	-	19	ribosome	2003
5	1P0L	-	19	ribosome	2003
6	1P0O	-	19	ribosome	2003
7	1OT0	-	19	antibiotic	2004
8	1YG0	HP1073	66	metal transport	2006
9	1ZHC	HP1242	76	hypothetical protein	2005
10	2LXR	HP1264	76	oxidoreductase	2012
11	2H9Z	HP0495	86	hypothetical protein	2007
12	1Z8M	HP0894	88	hypothetical protein	2005
13	2KI2	HP0827	90	RNA binding protein/ ssDNA binding protein	2009
14	2JOQ	-	91	hypothetical protein	2007
15	2K6P	HP1423	92	unknown function	2009
16	2OTR	HP0892	98	hypothetical protein	2007
17	2HQN	-	109	singnaling protein	2007
18	1X93	HP0222	110	transcription	2005
19	2K0Z	HP1203	110	unknown function	2008
20	2K4J	-	115	transcription	2008
21	2KDX	HP0869	119	metal binding protein	2009
22	2K1O	HP0564	132	gene regulation	2008
23	2KR7	HP1123	151	isomerase	2010
24	2HQO	-	246	singnaling protein	2007
25	2HQR	HP1043	446	singnaling protein	2007

### 3.1. Proteins under 20 Amino Acid Residues: 1P0O, 1P0L, 1P0J, 1P0G, 1P5L, 1P5K, 1OT0

The first structures determined by NMR were very small antimicrobial proteins with 19 amino acid residues in 2003 (PDB ID: 1P0G, 1P0J, 1P0L, 1P0O, 1P5K, and 1P5L) [32]. 1P0G is a 19-residue peptide derived from the N-terminus of *H. pylori* ribosomal protein L1, which has antimicrobial activity. Five analogues were made: 1P0J, 1P0L, 1P0O, 1P5K, and 1P5L, modified from 1P0G in order to explore their antimicrobial activities. 1P0J was made by substitution of 18Asp to Trp, 1P0L by 16Gln to Trp, 1P0O by 16Gln and 18Asp to 16 and 18Trp, 1P5K by 11Leu to Ser and 1P5L by 5Phe to Ser. 1OT0 has same sequence with 1P0J. These substitutions were based on the helical wheel-diagram for maintaining or raising the amphipathic characteristic, which plays an important role in antibiotic activity. Although they had amino acid substitutions at one or two points, they showed different hydrophobicities and antimicrobial activities. They all had just one helix structure in a SDS micelle environment.

### 3.2. Proteins between 20 and 99 Amino Acid Residues: 1YG0, 1ZHC, 2LXR, 2H9Z, 1Z8M, 2KI2, 2JOQ, 2K6P, 2OTR

1YG0, HP1073 (HPCopP) is a putative copper binding regulatory protein composed of 66 amino acid residues [33]. The small HPCopP is homologous to CopZ, encoded by the *E. hirae* and *B. subtilis* cop operons. CopZ proteins have a conserved structure, β-α-β-β-α-β with a similar metal binding region. However, the structure of HPCopP has a β-α-β-β-α fold (Figure 3A). The helices correspond to residues 14-23 (αI), and 51-60 (αII), while the sheets correspond to residues 2-5 (βI), 29-35 (βII), and 40-45 (βIII). Chemical shift perturbation experiments using samples with or without metal ions have revealed that the structure of HPCopP is not adequate for Cu(II) binding and seems to be optimized for the transfer of toxic Cu(I) resulting in copper trafficking through HPCopP by delivering Cu(I) to HPCopA [33]. The role of HpCopP in copper metabolism can be clarified from the structural and copper ion binding properties of HpCopP by NMR spectroscopy.

1ZHC, the HP1242 gene of *H. pylori*, encodes a 76-residue conserved hypothetical protein from *H. pylori* strain 26695 with a molecular weight of 9.1 kDa and a calculated pI of 6.1 [34,35]. The structure of HP1242 is fully helical, and is composed of three α-helices. These correspond to residues 6-14 (αI), 18-38 (αII), and 43-75 (αIII) (Figure 3B). Based on the sequence homology, this protein is classified as the Domain of Unknown Function (DUF) 465 family, which as indicated by the name, has an unknown function. These family members are found in several bacterial proteins, and also in the heavy chain of eukaryotic myosin and kinesin, which are predicted to form a coiled coil conformation.

2LXR, FMN-bound HP1264, could be involved in the initial electron transfer step of complex I (NADH-quinone oxidoreductase) [36]. HP1264 is structurally most similar to *E. coli* TusA which belongs to the SirA-like superfamily with an IF3-like fold in the SCOP database, implying that HP1264 adopts a novel folding for FMN binding. HP1264 is composed of 76 amino acid residues with a molecular weight of 8.9 kDa and has no conserved cysteine residues. HP1264 folds into a compact two-layered α/β-sandwich structure with the topology described as β-α-β-α-β-β, comprising a mixed four-stranded β-sheet stacked against two α-helices, both of which are nearly parallel to the strands of the β-sheet. The β-strands correspond to the residues 3-5 (βI), 28-35 (βII), 55-57 (βIII), and 68-74 (βIV), while the α-helices correspond to the residues 20-24 (αI) and 40-50 (αII). Based on the SCOP database, it is revealed that HP1264 adopts an IF3 (translation initiation factor 3)-like fold. However, structural motifs involved in the activities of the structural homologues are not conserved in this protein. HP1264 was identified as a novel FMN binding protein, based on the data from tryptophan fluorescence and NMR experiments. That means FMN-bound HP1264 could participate in the beginning of the electron transfer pathway of complex I from *H. pylori* (Figure 3C). Since HP1264 has a novel fold for RMN binding, it can be an antibiotic target of new mechanism.

2H9Z, the HP0495 gene of *H. pylori*, encodes an 86-residue hypothetical protein from *H. pylori* strain 26694 with a molecular weight of 10 kDa and a calculated pI of 8.71 [37,38]. The result of a sequence homology search showed that HP0495 has a restricted sequence homology with unknown proteins from several bacteria, except for the protein from *H. pylori* strain J99 (86% identity). Several proteins were identified with below 40% sequence identity with HP0495. HP0495 has two α-helices and four β-sheets (β-a-β-β-a-β: ferredoxin fold), and the β-strands form four-stranded anti-parallel β-sheet. The helices correspond to residues 28-34 (αI) and 66-76 (αII), while the β-strands correspond to residues 15-22 (β1), 41-44 (β2), 55-65 (β3), and 82-85 (β4) (Figure 3D).

1Z8M, HP0894, is an 88-residue, conserved hypothetical protein from *H. pylori* strain 26695 with a calculated pI of 8.5 and molecular weight of 10.38 kDa [39,40]. The HP0894 structure has two α-helices, two 3_10_-helices and four β-strands, namely α-α-3_10_-β-3_10_-β-β-β. The β-strands form four-stranded anti-parallel β-sheet (Figure 3E). The structural homology revealed HP0894 may have potential ribonuclease activity and represents the toxin-antitoxin (TA) system like RelE.

2KI2, HP0827 protein, is an 82-residue protein identified as a putative ss-DNA-binding protein 12RNP2 precursor from *H. pylori* [41]. It has a ferredoxin-like fold, β-α-β-β-α-β and ribonucleoprotein (RNP) motifs which are thought to be important in RNA binding. The β-strands correspond to residues 2-7 (βI), 30-33 (βII), 45-50 (βIII) and 73-76 (βIV), while the α-helices correspond to residues 15-26 (αI) and 53-61 (αII) (Figure 3F). The four strands are arranged in a right-handed twist and form an anti-parallel β-sheet that packs against the two α-helices. It contains a conserved structure pattern with an RRM motif that consists of four β-strands and two α-helices arranged in an α/β sandwich. Findings on the putative RNA-binding sites of HP0827 will be helpful to find the exact RNA-binding sites and patterns of HP0827.

The solution structure of 2K6P, HP1423, which has 84 amino acid residues, was determined by NMR. This protein is a conserved hypothetical protein from *H. pylori* [42]. The structure of HP 1423 is composed of five β-strands and three α-helices, α-α-β-β-β-β-α-β. The β-strands correspond to residues 28-30 (βI), 33-34 (βII), 44-49 (βIII), 56-60 (βIV), and 78-80 (βV), while the α-helices correspond to residues 3-10 (αI), 21-25 (αII) and 74-76 (αIII) (Figure 3G). According to the Pfam database, HP1423 belongs to the S4 (PF01479) superfamily. The S4 domain is a small domain consisting of 60-65 amino acid residues that probably mediates binding to RNA. The structure of HP1423 revealed the presence of the αL-RNA binding motif in the protein, which is a general feature of several RNA binding protein families. 

2OTR, HP0892, is a 90-residue protein with a calculated pI of 9.38 and molecular weight of 10.41 kDa [43,44]. The HP0892 structure has three α-helices and five β-strands arranging the β-α-α-β-β-β-β-α topology. The α-helices correspond to residues 8-21 (αI), 25-36 (αII), 86-89 (αIII), while the β-strands correspond to residues 3-5 (βI), 47-48 (β2), 57-62 (βIII), 65-72 (βIV), and 77-84 (βV) (Figure 3H). In the Pfam database, HP0892 belongs to the plasmid stabilization system protein family (PF05016: this family encompasses RelE/ParE). Structure and function studies revealed this protein also forms TA systems like HP0894.

### 3.3. Proteins between 100 and 200 Amino Acid residues: 2HQN, 1X93, 2K0Z, 2K4J, 2KDX, 2K1O, 2KR7

2HQN is the C-terminal effector domain of HP1043 (2HQR) with 109 amino acid residues [45]. This protein consists of an N-terminal four stranded anti-parallel β sheet (strands β6-β9) and a helix bundle with three α-helices (α6, α7, and α8). The β-hairpin between α6 and α7 enables close interactions with β10. The two helices comprised of α7 and α8 form a helix-turn-helix DNA binding motif.

1X93, HP0222, is a protein with 86 amino acid residues, which behaves as a dimer [46]. The structure has the ribbon-helix-helix fold characteristic of the transcription factors of the Arc/MetJ family, all of which bind DNA as higher order oligomers (Figure 3I). A DALI search produced several possible structurally related proteins from the Arc/MetJ family of transcription factors, all of which form stable dimers. The overall structure of the dimer resembles the proteins of the Arc/MetJ family, including Arc, MetJ, Mnt, ParG and CopG. It consists of an intertwined dimer forming a stable hydrophobic core. Each subunit consists of a β-strand formed by residues 33-36 and two α-helices formed by residues 41-51 (helix A) and 58-70 (helix B). The β-strands from the two subunits form an anti-parallel β-sheet (β-ribbon). The inter-subunit contacts are numerous and also include contacts between the helix A from one subunit and helix B from the second subunit. Based on biological experiments like gel filtration, crosslinking and DNA eletrophoretic mobility shift assays (EMSA), this protein might be involved in acid-response mechanisms or bacterium-epithelial cell interactions, implying that HP0222 can be a promising antibiotic candidate.

2K4J, *H. pylori* ArsS-ArsR two-component signal transduction system, comprised of a sensor histidine kinase (ArsS) and a response regulator (ArsR), allows the bacteria to regulate gene expression in response to acidic pH conditions [47]. Full-length ArsR protein and the DNA-binding domain of ArsR (ArsR-DBD) were expressed and purified, and the structure of the ArsR-DBD was solved. The structure of ArsR-DBD consists of an N-terminal four-stranded β-sheet, a helical core, and a C-terminal β-hairpin. The final topology of this protein is β-β-β-β-α-β-α-α-β-β (Figure 3J). The overall tertiary fold of the ArsR-DBD is most closely similar to the DBD structures of the OmpR/PhoB subfamily of bacterial response regulators. Because two-component systems are important for surviving in acidic environment, this tertiary structure can be helpful for understanding the adaptation of *H. pylori* in the stomach.

2KDX, HP0869, is coded by the hypA gene from *H. pylori* [48]. The zinc-bound HypA (Zn-HypA) exists as a monomer in solution and its solution structure was determined by NMR. Zn-HypA folds into two domains, including a zinc domain and a nickel with a mixed α/β structure. HypA and HypB also play an important role in the nickel maturation of urease in most *H. pylori* species. Zn-HypA folds into two domains, including a zinc domain and a nickel domain with a mixed α/β structure consisting of α-β-α-β-β-β-β-β topology. The nickel domain consists of 68 N-terminal and 10 C-terminal residues and contains a Ni-binding site. This domain folds into a globular structure encompassing a three-stranded anti-parallel β-sheet flanked on one side by two α-helices with a topology of α-β-α-β-β (Figure 3K). The zinc domain is formed by 33 residues containing two pairs of conserved CXXC motifs, which is a typical Zn-binding motif. Structural studies on metal-binding site of Zn-HypA can explain how to traffick metal ions and furthermore how to deliver nickel ions into the urease active site.

The solution structure of 2K1O, HP0564 from strain J99, is a protein with no assigned function [49]. Although it has no sequence homologs outside of *H. pylori*, its structural analysis indicates that it is a member of the ribbon-helix-helix superfamily (PF01402) of transcriptional regulators (Figure 3L). These proteins bind to specific DNA sequences with high affinity and usually act as repressors. The structure of HP0564 suggests that it may be a member of the ribbon-helix-helix (RHH) superfamily of transcriptional regulators. 

2KR7, HP1123, is a folding helper with both chaperone and peptidyl-prolyl cis-trans isomerase (PPIase) activities. The chaperone activity prevents the aggregation of unfolded or partially folded proteins and promotes their correct folding. PPIases catalyze the cis-trans isomerization of Xaa-Pro bonds of peptides, which accelerates the slow steps of protein folding and thus shortens the lifetime of intermediates. Both strategies lower the concentration of intermediates and increase the productivity and yield of the folding reaction. In addition, this protein is involved in hydrogenase metallocenter assembly, probably by participating in the nickel insertion step. This function in hydrogenase biosynthesis requires chaperone activity and the presence of the metal-binding domain, but not the PPIase activity. The N-terminal region consists of two globular folded domains that contain the prolyl isomerase and chaperone activities. The C-terminal region binds nickel ions, belonging to the FKBP-type PPIase family which contains 1 PPIase FKBP-type domain (Figure 3M).

### 3.4. Proteins over 200 Amino Acid Residues: 2HQO, 2HQR

2HQO is the N-terminal regulatory domain of HP1043 (2HQR) with 123 amino acid residues. This protein forms a compact dimer and the transactivation domain is connected to the regulatory domain by a short flexible linker. The molecular topology is an α/β-fold with a central five stranded β-sheet surrounded by five α-helices (Figure 3N). 2HQR, HP1043, is a new type of response regulator. This protein is a symmetric dimer, which consists of twelve β-strands and eight α-helices in each monomer unit. The molecular topology resembles that of the OmpR/PhoB subfamily with a 2-fold symmetry in the absence of phosphorylation. Through NMR spectroscopy, X-ray crystallography and site-directed mutagenesis, it can be presented how an atypical bacterial response regulator protein could function as a cell growth-associated regulator without a phosphorylation. That means it could possess regulator activity without phosphorylation, because the structure of the regulatory domain resembles that of the active, phosphorylated form of other well-known regulator protein.

## 4. Structure and Function Studies of HP0894 and HP0892

**Figure 4:**
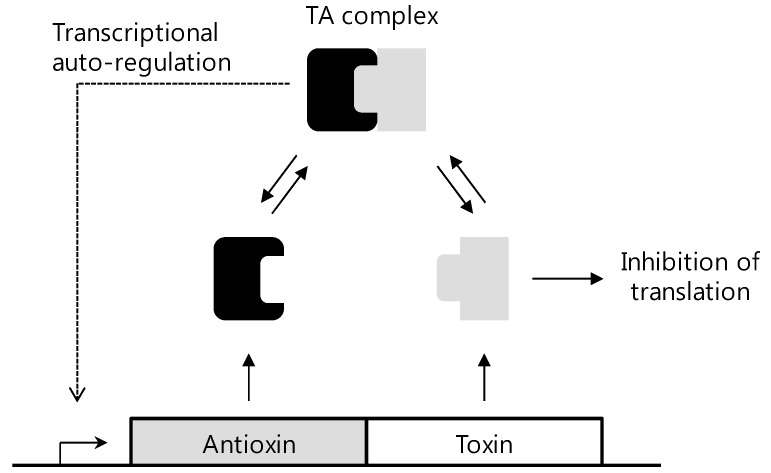
Mechanism of TA systems.

As mentioned above, HP0894 and HP0892 were hypothetical proteins. Based on the NMR structure determination, structural homologies were searched for using BLAST, Pfam, and DALI. From the results, it was found that HP0894 may have a potential ribonuclease activity and represent the toxin-antitoxin (TA) system like RelE [50] and HP0892 also acts as a TA toxin with intrinsic RNase activity [51]. In a TA system, the toxin molecules are negative regulators which are harmful to cell due to cleavage of DNA or RNA, transfer of phosphate groups, phorphaorylation of proteins, inhibition of ATP synthesis and so on [52,53,54,55]. The antitoxin molecules are positive regulators which bind toxins themselves either on a gene or protein level, and regulate the activity of the toxin. Thus, the expression levels and interactions between toxin and antitoxin are important in life of bacteria, so that TA systems are considered potent targets for the development of new antibiotics. Experiments like RNA cleavage, primer extension, gel filtration and NMR titration on HP0894 and HP0892 revealed that HP0894 acts as a toxin with HP0895 as an antitoxin, and HP0892 acts as a toxin with HP0893. TA system research on the structural studies of HP0894 and HP0892 introduces the possibility of a chromosomal TA system for which these two proteins may be relevant in the pathogenicity of *H. pylori*. In addition, because HP0894-HP0895 and HP0892-HP0893 are the only two TA pairs in *H. pylori* as known so far, the TA systems in *H. pylori* are related to the status of the infections of *H. pylori* in the human gastric mucosa, probably through negative regulation of the toxin molecules by the antitoxins. Figure 4 shows a brief schematic mechanism of TA systems. The toxin protein and antitoxin protein are encoded within a single operon, in which the toxin gene is usually located directly downstream of the antitoxin gene. The toxin expression induces the arrest of cell growth or cell death, while the antitoxin neutralizes the toxin by a direct protein-protein interaction [56]. Based on the structures of toxin proteins or antitoxin protein, binding site of toxin-antitoxin can be the good target for antibiotics design. For example, inhibitor of antitoxin can be structurally developed in order to enhance toxin activity resulting in cell death, or blocker of binding toxin-antitoxin can be designed to hinder binding of proteins. Otherwise, we could design new inhibitor in RNA levels. Thus, structural understanding of HP0894-HP0895 and HP0892-HP0893 will provide information of exact site of inhibitors or mechanism to inhibit in TA systems of *H. pylori*. Furthermore, because TA systems appear only in prokaryote and are directly related to cell death, TA systems like HP0894-HP0895 and HP0892-HP0893 may be promising targets for antibacterial agents. Therefore, the knowledge of the three dimensional structures of HP0894-HP0895 and HP0892-HP0893 TA systems can facilitate to develop new antibacterial agents for especially *H. pylori*. The method of determining structure and studying function using NMR can provide helpful information for the design and development of new antibiotic drugs.

## 5. Conclusions

Even though there are only 25 NMR structures of proteins from *H. pylori* to date, it has been proven that structural studies using NMR for antibiotics target discovery can be a more efficient method for small size proteins. NMR also makes it possible to study dynamics, intermediate states and so on. Studying the structures of proteins from *H. pylori* with NMR has been going on for just 10 years since 2003, while the study of structures with X-ray crystallography started two years earlier, mostly in 2001. The structures of *H. pylori* proteins determined by both X-ray and NMR correspond to just under 20% of the 1,600 genes which is the total number of *H. pylori* proteins. Moreover, structures determined only by using NMR are very few. That means more structures of *H. pylori* should be determined for antibiotics development for *H. pylori*. As seen in the examples of HP0894 and HP0892, the functions of *H. pylori* proteins have been revealed through NMR structural studies, which can help to find drug targets more efficiently and quickly. If we know the structures of the target sites, we can quickly modify the antibiotics conformation to overcome them when we discover drug resistant bacteria. Unfortunately, functional studies of the other proteins mentioned above are still going on. There are no antibiotics specialized for *H. pylori*, yet. However, these structural studies on *H. pylori* proteins will be the basis for antibiotics target discovery. Even though there are no antibiotics for *H. pylori* yet, drug discovery for new treatment is not so far into the future.
